# Differential Proinflammatory Signature in Vestibular Migraine and Meniere Disease

**DOI:** 10.3389/fimmu.2019.01229

**Published:** 2019-06-04

**Authors:** Marisa Flook, Lidia Frejo, Alvaro Gallego-Martinez, Eduardo Martin-Sanz, Marcos Rossi-Izquierdo, Juan Carlos Amor-Dorado, Andres Soto-Varela, Sofia Santos-Perez, Angel Batuecas-Caletrio, Juan Manuel Espinosa-Sanchez, Patricia Pérez-Carpena, Marta Martinez-Martinez, Ismael Aran, Jose Antonio Lopez-Escamez

**Affiliations:** ^1^Otology and Neurotology Group CTS495, Department of Genomic Medicine, Centre for Genomics and Oncological Research–Pfizer/Universidad de Granada/Junta de Andalucía (GENYO), Granada, Spain; ^2^Department of Pediatric Otolaryngology and Department of Orthopedics, The Feinstein Institute for Medical Research, Northwell Health System, Manhasset, NY, United States; ^3^Department of Otolaryngology, Hospital Universitario de Getafe, Getafe, Spain; ^4^Department of Otolaryngology, Hospital Universitario Lucus Augusti, Lugo, Spain; ^5^Department of Otolaryngology, Hospital Can Misses, Ibiza, Spain; ^6^Division of Otoneurology, Department of Otorhinolaryngology, Complexo Hospitalario Universitario, Santiago de Compostela, Spain; ^7^Department of Otolaryngology, Hospital Universitario Salamanca, Salamanca, Spain; ^8^Department of Otolaryngology, Instituto de Investigación Biosanitaria ibs.GRANADA, Hospital Universitario Virgen de las Nieves, Granada, Spain; ^9^Department of Otolaryngology, Hospital Universitario San Cecilio, Granada, Spain; ^10^Department of Otolaryngology, Complexo Hospitalario de Pontevedra, Pontevedra, Spain

**Keywords:** Vestibular Migraine, Meniere Disease, differential diagnosis, IL-1β, CXCL1, CCL3, CCL22

## Abstract

Vestibular Migraine (VM) and Meniere's Disease (MD) are episodic vestibular syndromes defined by a set of associated symptoms such as tinnitus, hearing loss or migraine features during the attacks. Both conditions may show symptom overlap and there is no biological marker to distinguish them. Two subgroups of MD patients have been reported, according to their IL-1β profile. Therefore, considering the clinical similarity between VM and MD, we aimed to investigate the cytokine profile of MD and VM as a means to distinguish these patients. We have also carried out gene expression microarrays and measured the levels of 14 cytokines and 11 chemokines in 129 MD patients, 82 VM patients, and 66 healthy controls. Gene expression profile in peripheral blood mononuclear cells (PBMC) showed significant differences in MD patients with high and low basal levels of IL- 1β and VM patients. MD patients with high basal levels of IL- 1β (MDH) had overall higher levels of cytokines/chemokines when compared to the other subsets. CCL4 levels were significantly different between MDH, MD with low basal levels of IL- 1β (MDL), VM and controls. Logistic regression identified IL- 1β, CCL3, CCL22, and CXCL1 levels as capable of differentiating VM patients from MD patients (area under the curve = 0.995), suggesting a high diagnostic value in patients with symptoms overlap.

## Introduction

Meniere's Disease (MD) is a syndrome characterized by attacks of recurrent vertigo associated with ipsilateral cochlear symptoms, such as fluctuating sensorineural hearing loss (SNHL), tinnitus or aural pressure (1, 2).

Vestibular Migraine (VM) is defined by the occurrence of episodic vestibular symptoms and a history of migraine, with a temporal association in at least 50% of the attacks (3). VM has an estimated population prevalence of about 1%, however with only medical history and symptomology it may be indistinguishable from MD at times (4–6).

It has been described that 8.4% of MD patients have headache compatible with migraine, during vertigo attacks (7). The major difference in the diagnosis criteria for these diseases are the auditory symptoms, which are necessary for the diagnosis of definite MD, however it has been reported that VM patients may have tinnitus during vertigo attacks, and also that 25% of migraine patients suffer from hearing loss (7, 8).

VM patients have a favorable response to anti-migraine drugs, which supports an underlying migraine mechanism. However, this evidence is insufficient as it is based on uncontrolled clinical case series, thus the apparent efficacy may be due to confounding factors, such as placebo response, spontaneous improvement and multiple drug effect (3). Moreover, a high prevalence of migraine in patients with MD has been previously described, suggesting a pathophysiological link between these diseases (8, 9).

Recently, the Meniere's disease Consortium identified five clinical subgroups in patients with unilateral and bilateral MD: Group 1 was defined by SNHL starting first in one ear and involving the second ear in the next months or years; Group 2 characterized by simultaneous hearing loss in both ears since the onset of the disease; Group 3 clustered familial MD; Group 4 associated with migraine in all cases and Group 5 was found in patients with an autoimmune disease in addition to MD (10, 11). Additionally, Frejo et al have observed two subgroups of MD patients, according to their IL-1β profile, patients with high levels of IL-1β (MDH) or patients with low levels of IL-1β (MDL), which may have different immune responses or functional states of the immune system (12).

Considering that the cytokine profile may allow to subgroup MD patients and the clinical similarity of MD and VM, we propose to investigate if the pro-inflammatory signature of these diseases may allow to distinguish these patients.

## Materials and Methods

### Human Subjects

This study included a total of 129 patients with definite MD, 82 patients with VM, and 66 healthy controls. Patients were diagnosed according to the diagnostic criteria of the Barany Society for MD (1) and VM (3). The experimental protocols of this study were approved by the Institutional Review Board in all participating hospitals and every patient signed a written informed consent. The study was carried out according to the principles of the Declaration of Helsinki revised in 2013 for investigation with humans.

### PBMC Isolation and Incubation

Peripheral blood was diluted 1:1 with 1 × PBS and disposed carefully onto the corresponding 25:15 volume of Lymphosep, Lymphocyte Separation Media (Biowest, Nuaillé, France). Samples were centrifuged for 20 min at 2,000 rpm to separate blood content. PBMC were collected and washed with 1 × PBS and cultured in RPMI 1640 supplemented with 10% (v/v) fetal bovine serum (Biowest, Nuaillé, France) and plated at 1.25 × 10^6^ cells/mL in 6-well plates. PBMC were incubated during 16 h at 37°C in 7% CO_2_. After the incubation, PBMC were centrifuged, RNA was harvested, and supernatants were collected and stored at −80°C.

### RNA Extraction and Expression Array

RNA was isolated using the High Pure RNA Isolation Kit (Hoffmann-La Roche, Basel, Switzerland) following the manufacturer's protocol. RNA concentration was measured on Nanodrop (NanoDrop Technologies Inc., Wilmington, DE, USA). RNA quality was checked using Agilent 2100 Bioanalyzer (Agilent Technologies, Waldbronn, Germany).

Expression levels were measured using the HumanHT-12 v4 Expression BeadChip (Illumina Inc., San Diego, CA, USA) with 500 ng of total RNA and processed with the high-resolution scanner iScan (Illumina Inc., San Diego, CA, USA).

The number of biological replicates for each condition was: 5 healthy controls, 4 MD patients with low cytokine basal levels, 3 MD patients with high cytokine basal levels and 6 VM patients.

Expression data analysis, normalization, differential expression analysis and Principal Component Analysis (PCA) was carried out as previously described by Frejo et al. (12). For pairwise comparisons fold-change>2 and adjusted *p*-value 0.05 cut-offs were used.

Core analysis was performed using Ingenuity Pathways Analysis (IPA®, Qiagen, Venlo, Netherlands, http://www.ingenuity.com/products/ipa) software, using the differentially expressed genes (DEG) with an adjusted *p*-value cut-off of 0.02.

### Cytokine Measurement

Supernatants were collected and stored at −80°C until enough samples were acquired. Frozen samples were thawed immediately prior to analysis and none of the samples underwent more than two freeze-thaw cycles prior to analysis. Fourteen cytokines (IFNα-2, IFN-γ, IL-10, IL-1α, IL-1β, IL-1rα, IL-4, IL-6, IL-12p40, IL-12p70, IL-13, IL-17, IL-28A, and TNFα), and 11 chemokines (IL-8, CXCL10, CCL2, CCL3, CCL4, CCL5, CD40LG, CXCL1, CCL22, CXCL5, and CCL8) were measured using the commercially available Multiplex Bead-Based Kits (EMD Millipore, Billerica, MA, USA). The measurements were done in accordance with the kit-specific protocols provided by Millipore, using a Luminex 200 (Luminex Corp., Austin, TX, USA) and read with Luminex x PONENT 3.1 software (Luminex Corp.). The minimum detection limit for the assays can be found in Table 1. Samples with readings below or above these levels were assigned values of 0 pg/mL for the minimum value or 10,000 pg/mL for the maximum value. Two quality controls for each cytokine were run in duplicate. As the levels of IL-12p40, IL-12p70, IL-13, IL-17, and IL-28A were low or undetectable among all groups, they were excluded from the statistical analysis.

**Table 1:** Assay sensitivity determined as Minimum Detection Concentration plus 2 Standard Deviations (MinDC+2SD) for the Milliplex® human cytokines/chemokines quantified.

**Cytokine**	**MinDC+2SD (pg/mL)**
IFNα2	4.8
INFγ	1.1
CXCL1	14.1
IL-10	1.6
IL-12p40	12.7
IL-12p70	1
CCL22	7.1
IL-13	1.9
IL-15	1.7
sCD40L	9.9
IL-17	1.2
IL-1RA	17.1
IL-1α	12.6
IL-1β	1
IL-4	7.1
IL-6	1.3
CXCL10	14
CCL2	3.4
CCL3	6.2
CCL4	4.8
TNFα	1.1
CCL5	1.9
CCL8	2.2
CXCL5	7.2
IL-28A	7.9

### Statistical Analysis

A descriptive analysis was conducted using SPSS software v.22 (SPSS Inc., Chicago, IL, USA) for the clinical data and displayed as mean ± standard error for the mean (SEM). Quantitative variables were compared using Mann-Whitney U test, Student's unpaired *T*-test and Krustal-Wallis H test. Qualitative variables were compared using Pearson Chi-square Test and Krustal-Wallis H test. The level of significance considered was *p*-value < 0.05.

Representative heatmaps of the cytokine levels were done using the gplots package for R.

### Logistic Regression

We identified cytokines with differential production between MD and VM using Mann-Whitney test. Next, we performed logistic regressions, removing step-by-step the variables which had higher *p*-values, until we obtained a model for which all cytokines were significant. The coefficient of determination *R*^2^, which summarizes the proportion of variance in the dependent variable associated with the predictor variables, was estimated by using Nagelkerke's *R*^2^. A receiver operating characteristic (ROC) curve was generated to determine the ability to predict VM based on a model composed of cytokine production. Area under the curves (AUC) was calculated for the ROC curves.

## Results

### VM Has an Earlier Onset Than MD

Table 2 compares the clinical features of 129 MD patients (26 MDH and 103 MDL) and 82 VM patients. Patients with VM were younger (*p* = 1.20 × 10^−5^) and had an earlier onset of the disease (*p* = 4.41 × 10^−4^) than MD patients. As expected, MD patients had worse hearing (*p* = 5.32 × 10^−22^), higher number of vertigo attacks (*p* = 2.87 × 10^−3^) and were more functionally affected by the disease (*p* = 1.44 × 10^−11^), when compared to VM patients. A significant difference was found in the prevalence autoimmune disease between VM and MD, namely MDL had the highest history of autoimmune disease (*p* = 9.77 × 10^−3^). On the other hand, VM patients suffered more from headaches (*p* = 4.43 × 10^−16^), and more specifically migraine episodes (*p* = 1.89 × 10^−26^) than MD patients. We also observed a higher number of drop attacks in patients with MDH (*p*-value = 2.24 × 10^−14^).

**Table 2:** Clinical and demographic variables assessed in patients with Meniere Disease with high levels of IL-1β (MDH), Meniere Disease with low levels of IL-1β (MDL), and Vestibular Migraine (VM).

**Variable**	**MDH (*N* = 26)**	**MDL (*N* = 103)**	**VM (*N* = 82)**	***p*-value**
Age (mean ± SD)	60.6 ± 11.2	59.3 ± 13.7	48.6 ± 16.0	**1.20** × **10**^**−5**^
Years of Evolution (mean ± SD)	12.0 ± 11.0	10.1 ± 8.5	10.9 ± 10.2	0.875
Age of onset (mean ± SD)	44.4 ± 10.5	47.3 ± 15.7	37.1 ± 17.2	**4.41** × **10**^**−4**^
Sex (% female)	50	63.6	65.1	0.366
Laterality (% unilateral)	50.8	63	NA	0.264
Affected ear (% right ear)	26.9	27.6	NA	0.321
Hearing loss (% synchronic)	30.4	18	NA	0.342
Time until evolving to bilateral (months)	125.0 ± 118.6	55.8 ± 59.9	NA	0.106
MD type				0.736
1	69.6	64.4	NA	
2	0	4.4	NA	
3	8.7	7.8	NA	
4	13	10	NA	
5	8.7	13.3	NA	
Hearing stage (%)				**5.32** × **10**^**−22**^
1	15.4	6.3	92.7	
2	26.9	29.2	7.3	
3	30.8	47.9	0	
4	26.9	15.6	0	
5	0	1	0	
Ear Family History (%)	12	29.4	34.9	0.130
Familial Meniere disease (%)	8.3	10.1	5,4	0.466
Headache (%)	38.5	37.6	98.6	**4.429 ×10–16**
Type of headache				**2.117** × **10**^**−7**^
Migraine	50	51.4	94.6	
Tensional	50	48.6	5.4	
Migraine (%)	20	20.5	100	**1.887 ×10**^**−26**^
Type of Migraine				0.569
Migraine with Aura	60	42.9	58.3	
Migraine without Aura	40	57.1	41.7	
History of autoimmune disease (%)	7.7	17.3	1.7	**9.77 ×10**^**−3**^
Tumarkin crises (drop attacks)	1.9 ± 0.3	1.6 ± 0.7	NA	**2.241 ×10**^**−14**^
Number of crisis in last 6 months	2.3 ± 3.0	2.0 ± 3.0	1.4 ± 2.1	**2.87 ×10**^**−3**^
AAO-HNS Functional level (1–6)				0.241
1	8	17.9	70.9	**1.44 ×10**^**−11**^
2	44	29.8	20	
3	16	27.4	9.1	
4	20	16.7	0	
5	12	6	0	
6	0	2.4	0	

*Significant differences for p-value < 0.05 are highlighted in bold*.

### Gene Expression Profile on PBMCs can Differentiate MD, VM, and Healthy Controls

We compared gene expression profiles of PBMCs from 6 VM patients, 7 MD patients [4 with low basal levels of IL-1β and 3 with high basal levels of IL-1β (12)] and 5 healthy controls.

**Figure 1:**
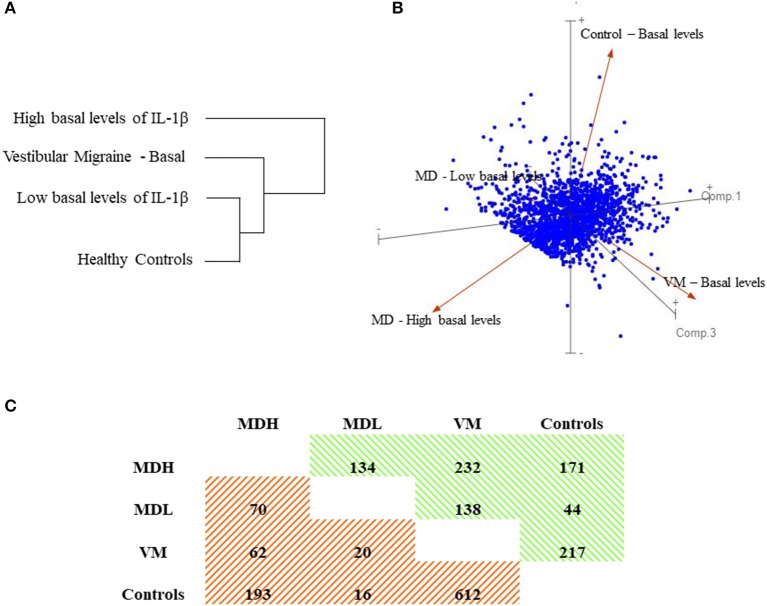
Gene expression in PBMC. **(A)** Dendrogram showing hierarchical clustering of patients and controls according to the gene expression in PBMC. **(B)** Three-dimensional loading plot showing principal component analysis (PCA) using all datasets. **(C)** Matrix of up- and down- regulated genes for selected pairwise comparisons. Values indicate the number of up- or down- regulated genes with a fold-change (FC) >2 and *P*-value < 0.02. In green are the up-regulated genes (positive FC) and in orange are the down-regulated genes (negative FC).

Using 1,894 genes, with 2 fold-change (FC) and an adjusted *p*-value = 0.05, the samples were clustered into 4 distinct groups by Principal Component Analysis (PCA), which corresponded to our 3 patient groups and healthy controls, as seen in Figure 1. Additionally, the hierarchical clustering of patients and controls according to the gene expression in PBMC, shows that MDL are more similar to healthy controls than to MDH.

**Figure 2:**
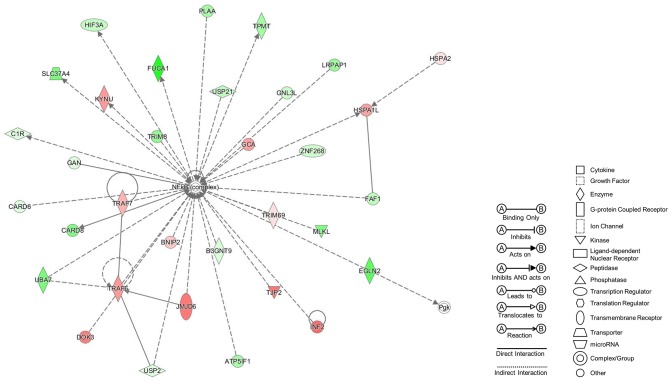
Top Network from VM patients PBMCs at basal levels. Network retrieved after comparing by pairwise analysis gene expression data from VM patients to healthy controls. Genes in red were up-regulated, while genes in green were down-regulated.

Pairwise comparisons between groups were carried out due to inter-individual variability. We firstly compared the gene expression profile in PBMC from patients with VM and healthy controls and found 832 DEG (*p*-value < 0.02 and FC>2; Supplementary Table 1), being the most significant gene *CACNA2D2* (-5.3 FC and adjusted *p*-value = 5.89 × 10^−6^). The core analysis with IPA software gave us a list of 25 networks. The top ranked network had a score of 37 and 30 focus molecules (Figure 2).

**Figure 3:**
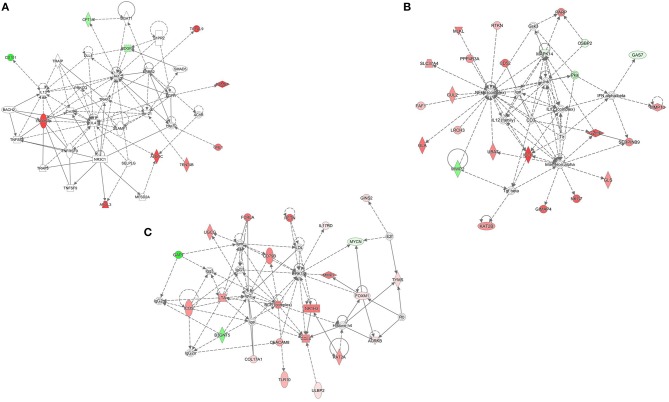
Differentially expressed genes comparing VM to MDH and MDL. **(A)** Network obtained after taking common genes from pairwise comparison between MDH and VM and MDL and VM. **(B)** Network retrieved in MDH patients unique differentially expressed genes (DEG), after pairwise comparison to VM. **(C)** Network retrieved in MDL patients unique DEG, after pairwise comparison to VM. Genes in red were up-regulated, while genes in green were down- regulated.

Additionally, we compared the gene expression profile in PBMC from patients with VM to MDH and MDL separately (Supplementary Tables 2, 3). We observed that 22 genes were differentially expressed in both comparisons, from which we retrieved a network with 10 focus molecules (score 23, Figure 3A). Of note, 286 genes were uniquely expressed when comparing PBMC from patients with VM to MDH, resulting in a top network with 22 focus molecules (score 32, Figure 3B). Moreover, 136 genes were uniquely expressed when comparing PBMC from patients with VM to MDL, resulting in a top network with 22 focus molecules (score 41, Figure 3C).

### VM Patients Have Similar Cytokine Levels to MD Patients With Low Basal Levels of IL-1β

The expression array revealed significant differences in various cytokines and chemokines, therefore we decided to measure the levels of IFNα-2, IFN-γ, IL-10, IL-1α, IL-4, IL-8, CXCL10, CCL2, CCL3, CCL4, CCL5, CD40LG, CXCL1, CCL22, CXCL5, and CCL8 in a smaller cohort of patients, which included 24 MDH patients, 24 MDL patients, 20 VM patients and 10 healthy controls. Levels of IL-1β, IL-1RA, IL-6, and TNFα were measured in all cases and controls, in line with previous work carried out by our group (12).

**Figure 4:**
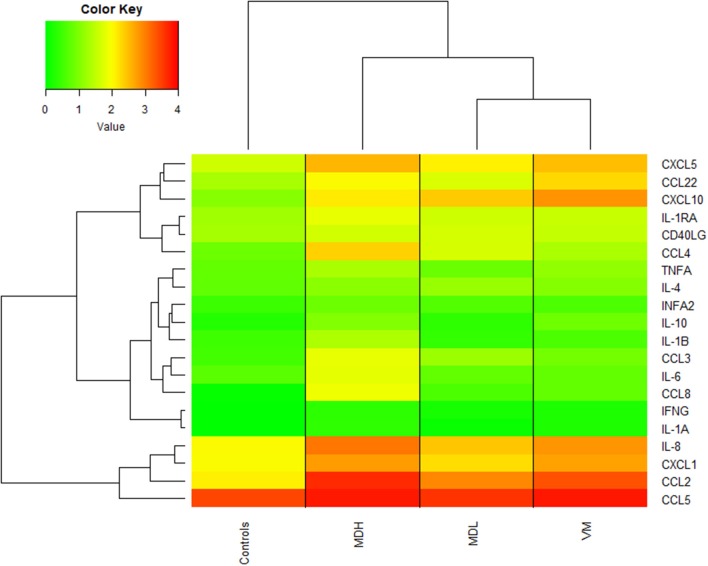
Heatmap of cytokine/chemokine concentrations [log_10_(concentration (pg/mL) +1)] with dendrogram showing hierarchical clustering of patients and controls.

The cytokines can be separated into 3 groups according to their levels: (a) high cytokine production in all groups, (b) cytokines which are higher in MDH than in the other groups and (c) cytokines that are higher in patients than in controls, as observed in Figure 4.

Also, we can observe that the cytokine levels of VM patients are very similar to MDL patients and that controls always have lower cytokine levels than the patients.

### CCL4 Levels May Allow to Distinguish Between MD, VM and Healthy Controls

MDL had the lowest number of cytokines that were statistically different from healthy controls, as no differences were found in the levels of IL-10, IL-1β, IL-6, CCL5, CXCL1, IFNA2, and CXCL5 (all, *p* > 0.1) (Supplementary Table 4). On the other hand MDH had statistically different levels of all cytokines comparing to healthy controls (*p* = 1.64 × 10^−12^-4.61 × 10^−2^].

Overall, MDH patients have higher levels of all cytokines, except for CCL22 (*p* = [2.12 × 10^−4^−3.01 × 10^−2^]) and CXCL10 (*p* = [2.18 × 10^−4^-2.10 × 10^−2^]), for which VM patients hold the highest levels.

We observed that CCL4 allows to distinguish between all groups of patients and between patients and controls (by pairwise comparison *p* = [6.30 × 10^−5^-1.04 × 10^−2^]). Moreover, CXCL10 allows to distinguish most groups *p* = [2.18 × 10^−4^-3.84 × 10^−2^]), however it does not discriminate MDH from MDL (*p* = 0.354).

Despite the high similarity of cytokine levels of VM patients and MDL patients, the levels of IL-10 (*p*-value = 9.84 × 10^−3^), CXCL1 (*p*-value = 1.42 × 10^−3^), and IL-8 (*p*-value = 9.79 × 10^−3^) are significantly higher in PBMC from VM patients than in MDL patients.

### IL-1β, CCL3, CCL22, and CXCL1 Levels May Allow to Distinguish Between MD and VM Patients

**Figure 5:**
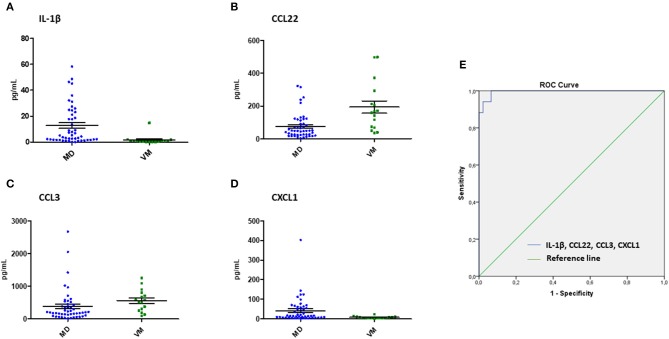
Predictive biomarkers for Vestibular Migraine. Scatter dot plots showing the measured levels for IL-1β **(A)**, CCL22 **(B)**, CCL3 **(C)**, and CXCL1 **(D)** in patients with VM and MD. **(E)** Receiver operating characteristic (ROC) curves to predict VM with IL-1β, CXCL1, CCL3, and CCL22. AUC (area under the curve) = 0.995.

In order to discriminate VM patients from MD patients, we performed logistic regressions with the differentially released cytokines between MD and VM, for which we obtained a final regression model that included IL-1β, CCL3, CCL22, and CXCL1 levels as independent variables (Table 3). The ability of the model to predict patients with VM was assessed by plotting a ROC curve using the variables from the logistic regression, which has a 93.8% sensitivity, 95.8% specificity, 97.8% positive predictive value and 11.8% negative predictive value (Figure 5). Other regression models using three, two or one of the cytokines were also evaluated, but all showed a lower diagnostic accuracy (Table 4).

**Table 3:** Logistic regression model to predict VM including IL-1β, CCL3, CXCL1, and CCL22 (Negelkerke's R^2^ = 0.909).

	**B**	**SE**	**Wald**	**Exp(B)**	**95% CI for EXP(B)**	***p*-value**
IL-1β	−1.99	0.94	4.50	0.14	0.02–0.86	0.03
CCL3	−1.07	0.45	5.68	0.34	0.14–0.83	0.02
CXCL1	0.02	0.01	5.22	1.02	1.00–1.04	0.02
CCL22	0.05	0.02	4.38	1.05	1.00–1.10	0.04

*The total accuracy of the model was 95.2%*.

**Table 4:** Different multivariant or univariant models using IL-1β, CXCL1, CCL3, CCL22 as predictive variables.

**Variables**	**AUC**	**% loss**
IL-1β, CXCL1, CCL22, CCL3	0.993	
IL-1β, CXCL1, CCL22	0.962	3.1%
CXCL1, CCL22, CCL3	0.948	4.7%
CXCL1, CCL22	0.794	21.0%
IL-1β, CCL22, CCL3	0.945	6.0%
IL-1β, CCL22	0.921	7.6%
IL-1β, CXCL1, CCL3	0.957	3.9%
IL-1β	0.874	11.9%
CXCL1	0.720	27.3%
CCL3	0.795	19.8%
CCL22	0.794	19.9%

*AUC–area under the curve*.

## Discussion

Our study shows that a small panel of cytokines and chemokines could be used for the differential diagnosis of VM and MD. Given that both conditions have a different therapeutic approach, our findings may contribute to improve the clinical management of VM and MD.

We observed that VM patients suffer more from migraine (*p* = 1.87 × 10^−26^) and have approximately a decade earlier onset than MD patients, (*p* = 1.20 × 10^−5^) and MD patients have more severe hearing loss (*p* = 5.32 × 10^−22^). Nevertheless, there is a symptom overlap which could be especially difficult to discriminate in earlier stages of the disease. Interestingly, MD type 4 patients, which are sporadic MD patients that suffer from migraine have been described to have a significantly earlier onset than the remaining MD patients (10, 11). These MD patients with comorbid migraine show migraine attacks that most of times are not associated with the episodic vertigo, but the temporal relationship between vertigo and migraine in MD needs to be investigated in a prospective longitudinal study. Specifically, bilateral MD type 4 (10) has the same mean age of onset (37 years old) as VM patients from our study, which further supports the necessity of a method to distinguish VM and MD patients that is not fully dependent of clinical information.

Our gene expression and cytokine results indicate that MDH and MDL are more similar between them than to VM. These results further support the hypothesis that MD is not a single disease or that it has various endophenotypes. Different MD subgroups have been already identified according to clinical manifestations and phenotype (10–12) and according to endolymphatic sac (ES) imaging (13).

Despite VM and MDL molecular similarity, these diseases seem to have distinct disease mechanisms, as there are 158 differentially expressed genes. When we compared the uniquely expressed genes between VM and MDL, we observe that the genes involved in the retrieved network are related to immune response, which seems to be upregulated in VM.

The presence of immunological activity in the inner ear has been previously described, both in animal models and humans. Immune responsiveness in the inner ear was firstly associated to the ES (14–16), however the presence of immune capacity in the cochlea has since been determined (17), namely due to the recent demonstration of IBA1 cells in the human ES and cochlea that express Major Histocompatibility Complex Type II (MHCII) (18). Nordström et al. (18) identified that the macrophage population of the stria vascularis, spiral ligament and spiral ganglion expressed MHCII, which is essential to initiate antigen-specific immune responses. Moreover, they propose that there might be an uptake and processing of antigens from the ES lumen, due to the co-expression of IBA1 and MHCII in epithelial cells and to trans-epithelial migration.

Here, we show that IL- 1β, CCL3, CCL22, and CXCL1 quantification could allow the differential diagnosis of VM and MD.

CXCL1, also known as GRO-α (Growth-related Oncogen-alpha) has an essential role in the recruitment and activation of neutrophils, mediating its function through CXCR2 signaling on neutrophils and by binding to glycosaminoglycans (GAG) on endothelial and epithelial cells and the extracellular matrix (19). CXCL1 is usually lower in patients with MD compared to VM.

CCL3 or MIP1- α (Macrophage Inflammatory Protein 1-alpha) is a macrophage secreted chemokine with inflammatory and chemokinetic properties, with CCR1, CCR4 and CCR5 binding. Recently, ES fibroblast cell lines, derived from MD patients have been established by Yamada et al. (20) and they observed that these cells produce CCL3 and that its production can be increased after Toll-like receptor (TLR) 3 and TLR4 stimulation. Moreover, they observed production of other cytokines, such as IL- 1β, CXCL10, thymic stromal lymphopoietin (TSLP), B lymphocyte stimulator (BLyS), IL-6 and IL-8. The levels of CCL3 are lower in patients with VM compared to MD, but higher than in healthy controls. Elevated levels of CCL3 have been reported in patients with migraine (21), which could support the hypothesis that VM shares migraine mechanisms (3).

CCL22 or MDC (Macrophage-derived chemokine) is a chemoattractant for monocytes, dendritic cells and natural killer cells and it binds to CCR4. IL-1β is a potent inflammatory mediator and is associated with various cellular mechanisms, such as cell activation, proliferation, and apoptosis (22). Yoshida et al cultured murine spiral ligament fibrocytes and verified that IL- 1β and TNFα stimulation resulted in production of various cytokines and chemokines, namely IL-6 and CCL2 (23).

The expression of CXCL1, CCL3, CCL22, and IL- 1β has been observed in mouse cochlear tissue (24), and CXCL1, CCL3, and IL-1β were also found in cells from the ES (25). Interestingly CXCL1 is the only chemokine which expression was also found in mouse vestibular cells (26) and of the four cytokines had the highest expression in all tissues.

Considering that we observed a cytokine increase in PBMCs extracted from peripheral blood, this could indicate that patients with MD and VM have a systemic proinflammatory response, which could explain some patients response to anti-inflammatory drugs (2, 27). On the other hand, this increased production of cytokines could also affect or represent activity that is occurring in the inner ear, thus the observed secretion of cytokines/chemokines may present a prolonged inflammatory response, leading to inner ear damage, through epithelial disruption.

Some studies have quantified cytokine levels in serum from migraine patients during the attack and non-attack periods. Fidan et al. (28) observed that IL-6 is higher in both periods when compared to healthy controls; CCL5, IL-10 and nitric oxide were only elevated during attack periods and TNFα, IL-1, IL-2, IFNγ, CCL2, CCL3, and CCL4 had no differences. On the other hand, Yucel et al. (29) saw an increase of CNDN5, ESM-1, IL-6, IL-1β, and TNFα during attacks. Additionally, Munno et al. (30) described an increase in TNFα, IL-4, and IL-5 in migraine patients, but no differences in IL-10 and IFNγ. In our work, we observed that VM patients had elevated levels of CCL5, IL-10, IL-1β, IFNγ, CCL2, CCL3, CCL4, and IL-4 (Supplementary Table 4) when compared to healthy controls, but no statistical differences in IL-6 and TNFα levels were found. Therefore, a comparison between VM patients and migraine patients, namely IL- 1β, CCL3, CCL22, and CXCL1 levels, could be beneficial to understand if there is a shared cytokine profile between migraine and VM or if this cytokine signature is specific to VM.

Autoinflammatory diseases are caused by a hyperactive inflammatory response, that leads to an immune deregulation, driven by IL-1, type I interferon, and NFκB (31). These diseases usually manifest themselves in the perinatal period, however milder and later-onset forms are being diagnosed in the adulthood (32). Some of these disorders have manifestations that mimic allergic and immunodeficiency disorders (31). Moreover, many of these diseases have sensorineural hearing loss as a symptom (31, 32). Autoimmune inner ear disease (AIED) is characterized by recurrent episodes of bilateral SNHL occurring over a period of several weeks or months (33). AIED has an overlapping audiovestibular phenotype with MD. Patients with AIED present elevated levels of proinflammatory cytokines, such as IL-1β and TNFα which resembles an autoinflammatory phenotype (33, 34). Recent efforts have been made to expand the immunological disease continuum to include monogenic and complex immune disorders that present both autoinflammatory or autoimmune manifestations, where diseases such as AIED would fall (33). Thus, considering the overall elevated levels of proinflammatory cytokines in patients with MDH and the similarity to AIED, we propose that MDH should be considered an autoinflammatory condition, yet longitudinal studies would be necessary to determine if these patients present continuously elevated proinflammatory cytokine levels or if it is a fluctuating condition.

In this study we were able to find a method that could allow a more accurate differential diagnosis between VM and MD. Since the cohort of patients for which we could measure all cytokines was limited (VM = 16 and MD = 48), these results should be replicated in a larger cohort of patients with VM, MD, and Migraine, also considering clinical variables to better characterize MD endophenotypes.

## Conclusion

Patients with MD or VM have a different pro-inflammatory signature. A cytokine panel including IL- 1β, CCL3, CCL22, and CXCL1 could be used as biological markers for the differential diagnosis of VM and MD.

## Data Availability

The datasets analyzed for this study can be found in the Gene Expression Omnibus with the accession number GSE109558: https://www.ncbi.nlm.nih.gov/geo/query/acc.cgi?acc=GSE109558.

## Ethics Statement

This study was carried out in accordance with the recommendations of the Declaration of Helsinki with written informed consent from all subjects. The protocol number PI17/1644 was approved by the Granada Ethical Review Board on the 29/01/18.

## Author Contributions

MF and LF performed experimental work including, PBMC isolation and culture, RNA extraction and cytokine measurement. MF, LF, AG-M performed statistical and bioinformatic analysis. EM-S, MR-I, JA-D, AS-V, SS-P, AB-C, JE-S, PP-C, MM-M, IA and JL-E recruited patients and obtained informed consent from all individuals. JL-E designed the study. JL-E supervised all experiments and MF and JL-E drafted the manuscript. All authors revised and approved the final version of the manuscript.

### Conflict of Interest Statement

Cytokine/chemokine panel for the differential diagnosis of episodic vestibular syndrome. Patent P201930255, March 20, 2019, owned by the Servicio Andaluz de Salud (Andalusian Public Health System). The authors declare that the research was conducted in the absence of any commercial or financial relationships that could be construed as a potential conflict of interest.
